# Time-Resolved DNA Stable Isotope Probing Links *Desulfobacterales*- and *Coriobacteriaceae*-Related Bacteria to Anaerobic Degradation of Benzene under Methanogenic Conditions

**DOI:** 10.1264/jsme2.ME13104

**Published:** 2014-06-06

**Authors:** Mana Noguchi, Futoshi Kurisu, Ikuro Kasuga, Hiroaki Furumai

**Affiliations:** 1 Research Center for Water Environment Technology, The University of Tokyo, 7–3–1, Hongo, Bunkyo, Tokyo 113–8656, Japan; 2 Department of Urban Engineering, Graduate School of Engineering, The University of Tokyo, 7–3–1, Hongo, Bunkyo, Tokyo 113–8656, Japan

**Keywords:** benzene degrader, DNA stable isotope probing (DNA-SIP), methanogenic enrichment culture, pyrosequencing, terminal restriction fragment length polymorphism (T-RFLP)

## Abstract

To identify the microorganisms involved in benzene degradation, DNA-stable isotope probing (SIP) with ^13^C-benzene was applied to a methanogenic benzene-degrading enrichment culture. Pyrosequencing of ribosomal RNA (rRNA) gene sequences revealed that the community structure was highly complex in spite of a 3-year incubation only with benzene. The culture degraded 98% of approximately 1 mM ^13^C-benzene and mineralized 72% of that within 63 d. The terminal restriction fragment length polymorphism (T-RFLP) profiles of the buoyant density fractions revealed the incorporation of ^13^C into two phylotypes after 64 d. These two phylotypes were determined to be *Desulfobacterales*- and *Coriobacteriaceae*-related bacteria by cloning and sequencing of the 16S rRNA gene in the ^13^C-labeled DNA abundant fraction. Comparative pyrosequencing analysis of the buoyant density fractions of ^12^C- and ^13^C-labeled samples indicated the incorporation of ^13^C into three bacterial and one archaeal OTUs related to *Desulfobacterales*, *Coriobacteriales*, *Rhodocyclaceae*, and *Methanosarcinales*. The first two OTUs included the bacteria detected by T-RFLP-cloning-sequencing analysis. Furthermore, time-resolved SIP analysis confirmed that the activity of all these microbes appeared at the earliest stage of degradation. In this methanogenic culture, *Desulfobacterales*- and *Coriobacteriaceae*-related bacteria were most likely to be the major benzene degraders.

Benzene is well known as a carcinogenic substance and has been categorized as Group 1 by the International Agency for Research on Cancer (International Agency for Research on Cancer, http://monographs.iarc.fr/ENG/Classification/). Benzene is readily degraded in the presence of oxygen. Although it was initially thought to be persistent in the absence of oxygen, Grbić-Galić and Vogel (13) reported the anaerobic mineralization of benzene in mixed methanogenic cultures. Recent studies demonstrated benzene degradation under other anaerobic conditions such as nitrate-, sulfate-, iron-, and carbon dioxide (CO_2_)-reducing conditions (33).

A few studies have examined pure cultures of benzene degraders under anaerobic conditions, *i.e.*, nitrate-reducing (6, 11, 18) and ferric iron-reducing (17, 36) conditions, but none have investigated these cultures under sulfate-reducing and methanogenic conditions.

Based on these findings, culture-independent molecular biological methods assisted by isotope tracer methods, such as stable isotope probing (SIP), are widely used to determine the phylogenetic and functional characteristics of uncultured microorganisms (23, 26, 27). The SIP method has recently been used to identify important microorganisms during the anaerobic degradation of benzene (8), including sulfate-reducing and methanogenic degradation. By applying SIP with terminal restriction fragment length polymorphism (T-RFLP), Oka *et al.* (24) showed that a bacterium from the *Desulfobacteraceae* family was clearly involved in anaerobic benzene degradation under sulfate-reducing conditions and also possibly under methanogenic environments. Time-resolved SIP revealed that two phylotypes of bacteria, the *Cryptanaerobacter*/*Pelotomaculum* group of *Peptococcaceae* and *Epsilonproteobacteria*, incorporated the ^13^C derived from the ^13^C-labeled benzene (16). Under methanogenic conditions, Sakai *et al.* used SIP with denaturing gradient gel electrophoresis (DGGE), and identified *Syntrophus*-related bacteria, named Hasda-A, as putative benzene degraders in a methanogenic culture enriched from the non-polluted soil of a lotus paddy field (29). However, more precise investigations are needed on the bacteria involved in the initial degradation of benzene, especially under methanogenic conditions.

The objectives of this study were (1) to analyze precisely the microbial community structure of the enriched benzene-degrading culture, (2) to detect the microorganisms involved in the anaerobic mineralization of benzene, and (3) to identify the microorganisms responsible in the early step of benzene degradation under methanogenic conditions. The methanogenic benzene-degrading microbial community was characterized by pyrosequencing the archaeal and bacterial 16S rRNA gene. The microorganisms involved in benzene degradation were then detected and identified by DNA-SIP-based methods. T-RFLP and nearly complete 16S ribosomal RNA (rRNA) gene sequencing of the fragments were also used to identify the major microorganisms involved in methanogenic benzene degradation. We also applied pyrosequencing to time course samples of the heavy fractions of the labeled treatment and estimated the first microorganism that assimilated the bacteria. In order to investigate the commonality of the microorganisms involved in benzene degradation, uncontaminated river sediment that was geographically and geologically different from the site of our previous study (29) was used as an inoculum for enrichment.

## Materials and Methods

### Culture enrichment

The culture was enriched from uncontaminated river sediment collected from Shinshibakawa river in Saitama Prefecture, Japan (November, 2007). In each series, 10 g of sediment was mixed with 20 mL of sterilized ultrapure water in a 72 mL glass vial in an anaerobic glove box (Miwa, Osaka, Japan). The bottles were sealed using Teflon-coated butyl rubber stoppers (Maruemu, Osaka, Japan) and aluminum crimp caps (Maruemu). To create anaerobic conditions, the headspace of the vial was purged with CO_2_:N_2_ = 1:4 mixed gas and Na_2_S and L-cysteine were then added at a final concentration of 0.3 g L^−1^, as described by Masumoto *et al.* (22). The initial concentrations of benzene were set to 1.3 × 10^−3^, 1.3 × 10^−2^, 1.3 × 10^−1^, and 1.3 mM (*n* = 3, for each). To produce an abiotic control, an additional vial was prepared as a sterile control for each initial benzene concentration by autoclaving at 121°C for 60 min on three d. The culture was incubated at 25°C without shaking. After 122 d, the triplicates were combined in a 135 mL vial, and the incubation was continued.

Accompanied by the production of CH_4_, the benzene concentration was firstly below the detection limit (0.3 μM) on day 250, while the sterilized control showed no benzene decrease and no methane production. Benzene was then repeatedly added via an anoxic benzene saturated aqueous solution at a concentration of approximately 1.3 × 10^−1^ to 1.3 mM whenever benzene became depleted. The cultures in all vials were combined on day 340 and the incubation was continued until the beginning of the SIP experiment (1,283 d).

### Chemical analysis

The benzene concentration in the liquid phase was determined on the basis of Henry’s coefficient from the headspace concentration measured by gas chromatography using a flame ionization detector (GC–FID/GC2010, Shimazdu, Kyoto, Japan). ^13^C-labeled CH_4_ and CO_2_ concentrations were analyzed by GC–mass spectrometry (GC–MS/GCMS-QP2010, Shimadzu) by selecting the ion monitoring (SIM) mode. The analytical method was the same as that described by Masumoto *et al.* (22). In the CO_2_ analysis, 0.5 mL of slurry was sampled in a serum bottle and the headspace gas was analyzed after dissolved CO_2_ in the culture was purged by adding 0.1 mL of 1 M HCl.

### Amplicon pyrosequencing of community DNA in the enrichment culture

Amplicon pyrosequencing was applied to the total DNA extracted from the enrichment culture on day 1,458. DNA was amplified using universal primers targeting the V4 region of the 16S rRNA genes of both bacteria and archaea. The forward primer was 519F, the consensus sequence of *Escherichia coli* 16S rRNA gene position 519 (Noda and Sekiguchi, personal communication). Regarding the reverse primer, two primers 802R-4 (5′-TACNVGGGTATCTAA TCC-3′) and 802R-2 (5′-TACCAGAGTATCTAATTC-3′) were mixed at a molar ratio of R-4:R-2 = 1:0.1. These primers were extended with linker (adopter A, 5′-CCATCTCATCCCTGCGTG TCTCCGAC-3′, for the forward primer, adopter-B, 5′-CCTATC CCCTGTGTGCCTTGGCAGTC-3′, for the reverse primer), key (5′-TCAG-3′), and multiplex identifiers (MID). A unique six base sequence was designed for each sample for MID. The PCR reaction mixture contained 1 μM of each primer, 0.8 mM dNTP mix, 1.5 mM MgCl_2_, 1.25 units AmpliTaq Gold^®^ DNA Polymerase, LD, and 1×Gold PCR Buffer in 50 μL. PCR amplification was performed using a T3 Thermal Cycler (Biometra, Goettingen, Germany) or Veriti^®^ (Applied Biosystems, CA, USA) under the following conditions: initial denaturation at 95°C for 9 min; 30 cycles at 95°C for 45 s, 50°C for 45 s, and 72°C for 1 min; and a final extension at 72°C for 5 min. The sample amplicons were purified and concentrated using a MultiScreen PCRμ96 Filter Plate (Merck Millipore, MA, USA) and then mixed with four other samples (not related to this research), *i.e.*, 300 ng of each. The mixture was sent to Macrogen Japan (Tokyo, Japan) and analyzed in 1/8 region of a picotiter plate using the Roche 454 FLX Titanium platform.

Denoising of the primary data and construction of operational taxonomic units (OTUs) were performed using Quantitative Insights Into Microbial Ecology (QIIME) ver.1.5.0 on VirtualBox (5, QIIME Team, 2011, http://qiime.org/). Reads with a quality score <25, length <100 base and >390 base, and containing a homopolymer ≥6 base were initially eliminated from subsequent processing. The sequencing noise characteristics of pyrosequencing were removed by flowgram clustering (28). Denoised reads were clustered using uclust (12), and OTUs were grouped with 97% similarity. The representative sequence of an OTU was the most abundant sequence within each OTU. Each OTU was assigned to taxonomy by blast (1) on the basis of the 16S Greengenes rRNA gene data file released on February 4, 2011 (http://greengenes.lbl.gov/). OTUs were aligned using PyNAST (4) with the Greengenes core reference alignment (9). Chimeric sequences were eliminated using ChimeraSlayer (14).

### Stable isotope labeling of DNA, ultracentrifugation, and fingerprinting of the density-resolved nucleic acids

Cultures that had been enriched for 1,283 d were used in the SIP experiment. The cultures were prepared under three conditions: (i) ^13^C (labeled) treatment (with 99% ^13^C_6_-benzene; Cambridge Isotope Laboratories, Inc., MA, USA), (ii) unlabeled treatment (with ^12^C_6_-benzene; Kishida Chemical, Osaka, Japan), and (iii) sterilized control treatment (with ^12^C_6_-benzene; Kishida Chemical). In each series, 20 mL of the enrichment culture was incubated in a 72 mL brown glass vial. The vial for the sterilized control was autoclaved at 121°C for 20 min on three occasions. Three replicates were prepared for conditions (i) and (ii) and one for condition (iii). Each treatment was statically incubated for 64 d at 25°C. The benzene-saturated aqueous solution prepared anoxically was added to each vial to make a final benzene concentration of 0.8–1.0 mM on day 0. Headspace gas samples were collected every 3–6 d to monitor the degradation of benzene and production of methane and CO_2_.

Depending on the level of benzene degradation (approximately 0%, 33%, 66%, and 100% of the initial amount degraded on day 0, 23, 38, and 64, respectively), 1 mL of slurry was collected through the rubber stoppers using disposable syringes from each vial of the ^13^C-treatment and unlabeled treatment in the anaerobic glove box. The vials were mixed well before sampling. Total DNA was extracted from 1.0 mL of soil slurry using ISOIL for Beads Beating (Nippon Gene, Tokyo, Japan) with 20 mg of skim milk (BD Difco^TM^, MD, USA; Le Pont de Claix, France) (30). Three DNA extracts from the same treatment on the same day were combined for subsequent analyses.

The combined DNA was fractionated using equilibrium density gradient centrifugation and gradient fractionation, according to the method of Neufeld *et al.* (23). The centrifugation medium was prepared by loading total DNA (500 ng) onto a CsCl/gradient buffer (0.1 M Tris–HCl, 0.1 M KCl, 1 mM EDTA) to produce a total DNA/media solution volume of 5 mL. The tube containing DNA/media was processed by ultracentrifugation (45,000 rpm, 20°C, 72 h) to separate DNA according to its buoyant density.

The fractions were separated by carefully collecting DNA/media from the bottom of the tube after ultracentrifugation. Twenty-two fractions were collected from each DNA sample. The refractive indices of each fraction were measured using an AR200 Automatic Digital Refractometer (Reichert Technologies, NY, USA), and the buoyant density was calculated according to the equation d = αn – β, where d is the buoyant density of the solution, n is the refractive index of the solution, and α and β are constants with the values 10.927 and 13.593 at 20°C, respectively (3). The DNA samples were then re-precipitated using polyethylene glycol (PEG) solution (30% PEG 6000, 1.6 M NaCl) and dissolved in Tris–EDTA (TE) buffer (10 mM Tris–HCl [pH 8.0], 1 mM EDTA [pH 8.0]). Each DNA fraction was used in the subsequent analyses.

To detect benzene-assimilating bacteria, T-RFLP analysis was performed on each fraction of the labeled and unlabeled treatments of days 23, 38, and 64. The PCR primer set Ba27f-FAM (5′-FAMAGAGTTTGATCMTGGCTCAG-3′)/Ba907r(5′-CCGTCAATTC MTTTRAGTTT-3′) was used to amplify the partial bacterial 16S rRNA gene. *Hha* I-digested amplicons were desalted using the QIAquick PCR Purification Kit (Qiagen, Hilden, Germany). The size separation of terminal restriction fragments (T-RFs) was performed using an ABI Prism 3100 Genetic Analyzer (Applied Biosystems). GeneScanTM 1200 LIZ^®^ (Applied Biosystems) was used for the size standard.

The analysis was performed through GeneMapper^®^ ver. 4.0 (Applied Biosystems). Of all the peaks detected, those with a peak area smaller than 50 arbitrary units were eliminated from subsequent analyses. Fragments with lengths of 20 to 600 bases were used in subsequent analyses. The area of each peak was normalized by dividing its area with the total area of all the valid peaks in the same fraction.

By comparing the T-RFLP profiles of the fractions with the same or closest buoyant density of the labeled and unlabeled treatments, T-RFs that appeared prominently in the labeled treatment, but not in the unlabeled treatment were detected. The fractions with a buoyant density closest to 1.732 g cm^−3^, in which the difference in the T-RFs in the two treatments was maximized, were defined as “heavy” fractions.

### Sequencing of the heavy fractions

To determine the taxonomic classification of the T-RFs that were increased in the heavy fraction in the labeled treatment, almost full-length bacterial 16S rRNA genes in one of the heavy fractions (d = 1.729 g cm^−3^) of the ^13^C-treatment on day 64 were amplified and cloned. The primer set of 27f (5′-AGAGTTTGATCMTGGCTC AG-3′) and 1492r (5′-TACGGYTACCTTGTTACGACTT-3′), and the QIAGEN PCR Cloning^PLUS^ Kit (Qiagen) were used for amplification and cloning, respectively. The cloned 16S rRNA genes were subjected to T-RFLP analysis to identify clones with the same fragment length of the presumptive benzene-assimilating bacteria. Samples were sent to Fasmac Co. for the sequence reaction using BigDye Terminator V3.1 (Applied Biosystems) and sequenced using Applied Biosystems 3130xl/3730xl. The determined sequences were assigned to their appropriate taxonomy using the online Nucleotide BLAST program (BLASTn) at the National Center for Biotechnology Information (NCBI, http://blast.ncbi.nlm.nih.gov/Blast.cgi). The Nucleotide collection (nr/nt) or 16S ribosomal RNA sequences (Bacteria and Archaea) were used as the database search set. Using Molecular Evolutionary Genetics Analysis (MEGA) ver. 5.05 or 5.10 (31, http://www.megasoftware.net/), the phylogenetic tree was drawn from the clone sequences and highly relative sequences from the databases shown above. The fragment lengths of these sequences were calculated *in silico* using BioEdit version 7.2.5. (15).

To detect DNA sequences that were enriched in the heavy fractions only in the labeled treatment, the heavy fractions of the labeled and unlabeled treatments on days 23, 38, and 64 of the SIP incubation were subjected to amplicon pyrosequencing. The primer sequences, target region, and amplification conditions were the same as those explained in the community analysis. After purification and concentration, 100 ng from each of the six fractions were combined with five other samples (not related to this research), and analyzed in 1/8 region of a picotiter plate.

### Nucleotide sequence accession numbers

The nucleotide sequences determined in this study have been deposited under DDBJ accession numbers AB771440–AB771446 for the 16S rRNA genes of the clones SHRa-h11, SHRa-h17, SHRa-h36, SHRa-h65, SHRa-h06, SHRa-h23, and SHRa-h59, respectively; AB771447–AB771449 for the 16S rRNA genes of OTU_1012, OTU_2694, and OTU_3242, respectively; and AB771712 for the 16S rRNA genes of OTU_2071.

## Results

### Microbial community of the enrichment culture

Amplicon pyrosequencing was performed using total DNA extracted on day 1,458 from the benzene-degrading enrichment culture. In total, 15,199 reads were obtained: 96.6% bacteria and 3.1% archaea. The major phyla were *Firmicutes (*34.6% of the bacterial reads), *Proteobacteria* (23.6%), and *Chloroflexi* (9.1%), which comprised 67.3% of the bacterial reads (Table 1). *Deltaproteobacteria* was the most abundant class (76.9%) among the *Proteobacteria*.

The most abundant taxonomic assignment was unclassified *Clostridiales*, which accounted for 27.8% of the bacterial reads (Table 1). Within the unclassified *Clostridiales*, a single OTU constituted 26.3% of the bacterial reads. All of the other OTUs had abundances of <5% (Table 1), and over half of all reads could not be assigned to defined families. These undefined OTUs included a large number of reads in candidate phyla, such as OP1, OP3, and OP8, from which no isolates had previously been obtained. When the top OTU of unclassified *Clostridiales* was processed by RDP Classifier (7, 35, Ribosomal Database Project, http://rdp.cme.msu.edu/classifier/classifier.jsp) with a confidence threshold of 50%, the sequence was classified as the genus *Eubacterium* under the family *Eubacteriaceae*.

### Degradation of benzene to CH_4_ and CO_2_

**Fig. 1:**
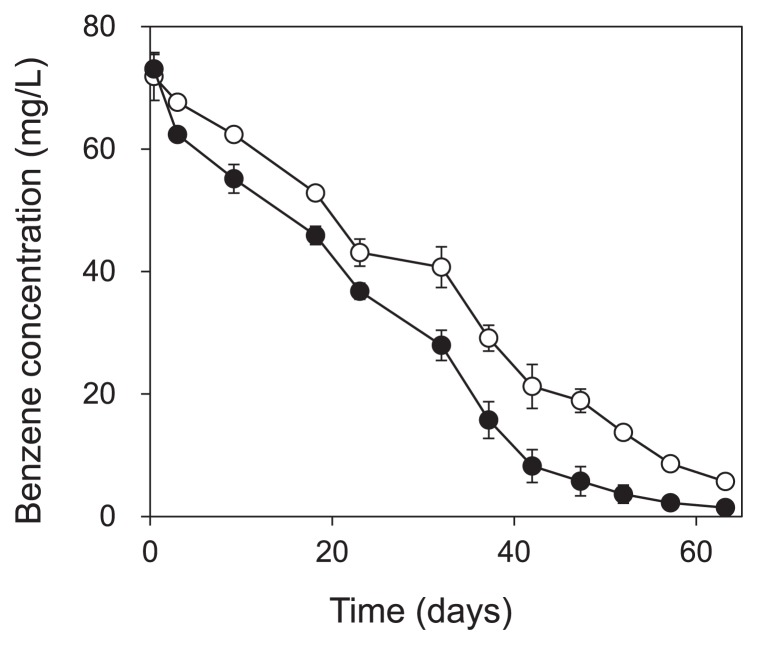
Concentration of benzene during incubation of the ●: labeled treatment, and ○: unlabeled treatment. Error bars show the standard error for triplicate samples.

**Fig. 2:**
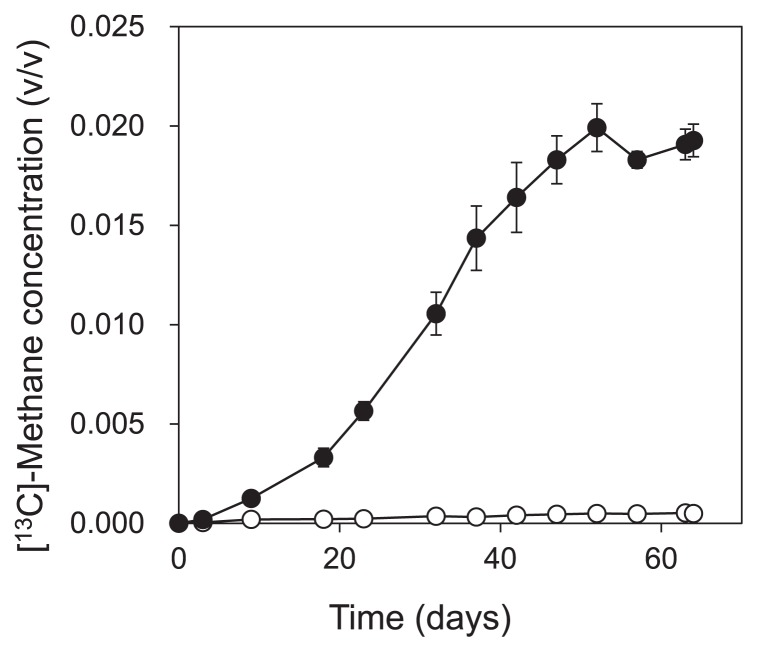
^13^C-methane concentration during incubation of the ●: labeled treatment, and ○: unlabeled, and ●: labeled treatment. Error bars show the standard error for three replicates in each treatment.

Part of the enrichment culture was used in the SIP experiment for 64 d. Of the benzene added on the first day of the SIP experiment, we found that 98% and 91% of ^13^C-benzene and unlabeled benzene, respectively, were degraded within 63 d (Fig. 1). CH_4_ and CO_2_ production was observed immediately after the degradation of benzene started (for CH_4_, Fig. 2). The amount of ^13^C-benzene degraded in the labeled treatment was 157.7 ± 23.7 μmol-C vial^−1^, and 44.1 ± 1.9 μmol-C vial^−1^ of ^13^CH_4_ and 68.8 ±1.0 μmol-C vial^−1^ of ^13^CO_2_ were produced.

### Detection of benzene-assimilating bacteria by DNA-SIP

DNA was extracted at different stages of the benzene degradation (day 0, 23, 38, and 64) in the batch culture incubated with labeled and unlabeled benzene, which was subjected to ultracentrifugation for density separation.

**Fig. 3:**
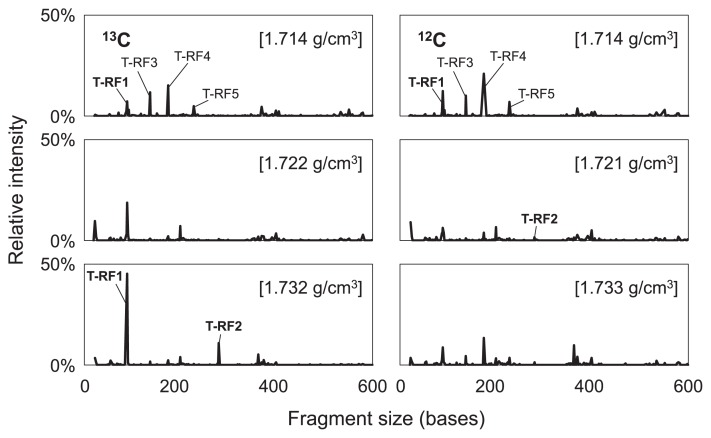
Terminal restriction fragment (T-RF) profiles of light, medium, and heavy fractions of the labeled (left column) and unlabeled (right column) treatments on day 64. Numbers in parentheses indicate the buoyant density of the fraction. Major peaks are numbered in the figures.

To screen the bacteria that incorporated ^13^C, the T-RFLP patterns of each fraction in the labeled and unlabeled treatments were compared on day 64 (Fig. 3). No significant differences were observed in the T-RF profiles of the “light” fraction (d = 1.714 g cm^−3^) between the labeled and unlabeled treatments, which contained T-RF1, T-RF3, T-RF4, and T-RF5 as the major fragments. In contrast, the T-RF profiles differed in the heavy fractions (d = 1.732 g cm^−3^) of the labeled and unlabeled treatments, in which two fragments, namely T-RF1 and T-RF2 in Fig. 3, became dominant in the labeled treatment, but not in the unlabeled treatment.

**Fig. 4:**
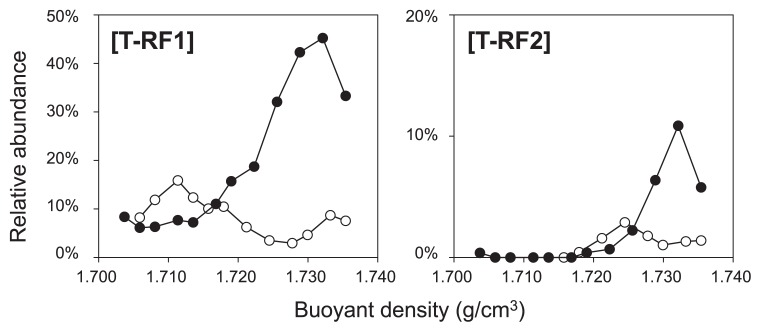
Relative abundance of T-RF1 and T-RF2 in each fraction of the ●: labeled treatment, and ○: unlabeled treatment on day 64. The *x*-axis shows the buoyant density (g cm^−3^) of each fraction.

The 10 most abundant T-RFs in the heavy fraction of the labeled treatment were selected, and the relative intensity of each fragment was compared in each fraction. Compared with the unlabeled treatment, only T-RF1 and T-RF2 exhibited a clear shift toward the heavier fractions of the labeled treatment (Fig. 4). Thus, the phylotypes of T-RF1 and T-RF2 appeared to be involved in methanogenic benzene degradation in this enrichment culture. Furthermore, the T-RFs that exhibited a clear increase in the heavier fractions of the labeled treatment on day 64, *i.e.*, T-RF1 and T-RF2, first increased on day 38 (Fig. S1).

### Phylogenetic assignment of ^13^C-benzene assimilating bacteria

**Fig. 5:**
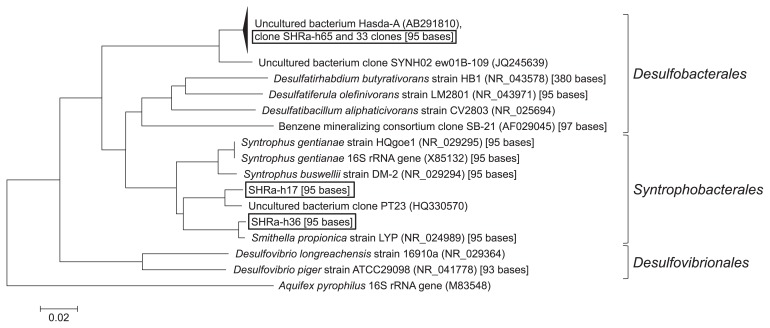
Phylogenetic tree of the bacterial 16S ribosomal RNA (rRNA) gene clones belonging to T-RF1 in the 1.729 g cm^−3^ fraction of the labeled treatment on day 64. Boxed clones are those collected in this study. The number in the parentheses indicates the accession number of the reference sequence. The number in the brackets indicates the T-RF length obtained by *in silico* analysis.

**Fig. 6:**
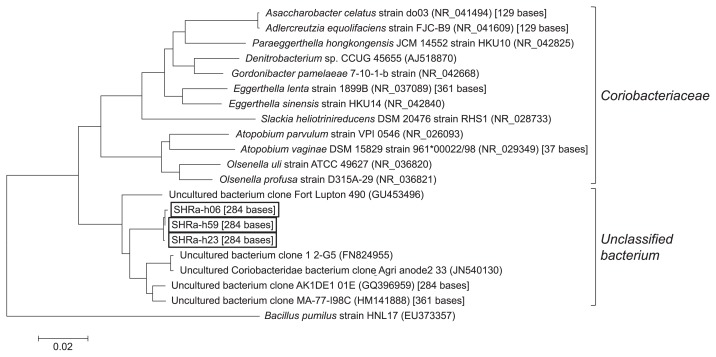
Phylogenetic tree of the bacterial 16S ribosomal RNA (rRNA) gene clones belonging to T-RF2 in the 1.729 g cm^−3^ fraction of the labeled treatment on day 64. Boxed clones are those collected in this study. The number in the parentheses indicates the accession number of the reference sequence. The number in the brackets indicates the T-RF length obtained by *in silico* analysis.

To determine the taxonomy of the phylotypes of T-RF1 and T-RF2, 76 clones were collected from one of the heavy fractions (1.729 g cm^−3^) on day 64. Of these, 36 clones had the same fragment length with T-RF1 and three clones with T-RF2. They had 95 bases and 284 bases T-RFs by *in silico* analysis, respectively. Most clones (34 out of 36 clones) of T-RF1 had >99% similarity to Hasda-A (29), the putative benzene-degrading bacteria in a methanogenic benzene-degrading enrichment culture, which originated from a different source, *i.e.*, lotus field soil. These clones (SHRa-h65 and 33 clones) phylogenetically belonged to the order *Desulfobacteriales* together with Hasda-A, which does not include the genus *Syntrophus* (Fig. 5). The remaining two clones, SHRa-h17 and SHRa-h36 were close to *Smithella propionica*, with sequence identities of 93% and 99%. On the other hand, the closest species to the T-RF2 phylotype was *Olsenella uli* in the family *Coriobacteriaceae*; however, its homology was only 89% (Fig. 6).

### Pyrosequencing of the heavy fraction

**Fig. 7:**
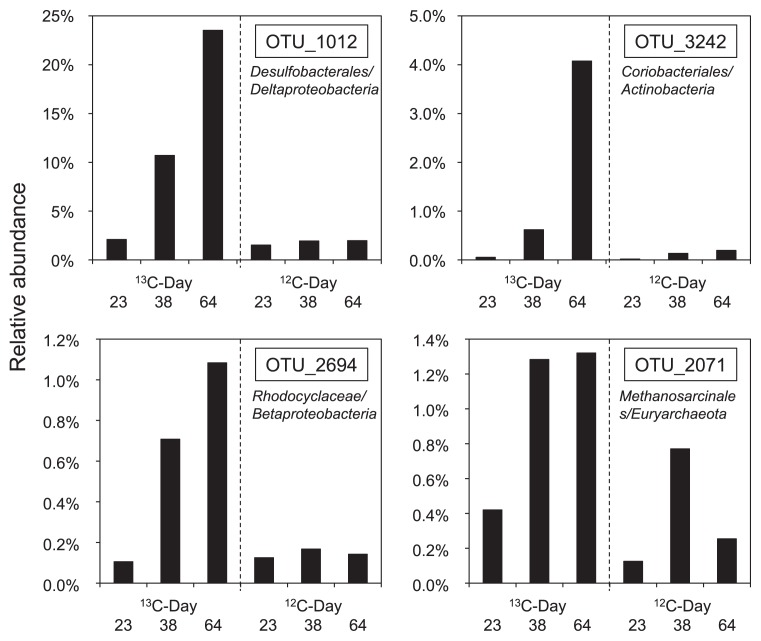
Relative abundance of operational taxonomic units (OTUs) that differed in their relative abundance between the labeled and unlabeled treatments in time-resolved stable isotope probing (SIP).

To more intensively analyze differences between the labeled and unlabeled treatments, we applied barcoded amplicon pyrosequencing to the heavy fraction of each treatment and compared the labeled and unlabeled treatments. We also extended this analysis to time-resolved SIP by analyzing samples after benzene degradation was nearly complete (day 64) and also during its degradation (on days 23 and 38). An average of 7,307 sequence reads (minimum = 1,904, maximum = 17,723) were obtained by pyrosequencing. Among the OTUs with a relative abundance of >1% in the heavy fraction of the ^13^C-treatment on day 64, a clear differences was only detected in the abundances of 4 OTUs between the labeled and unlabeled treatments. Regarding bacteria, the relative abundances of OTU_1012, OTU_3242, and OTU_2694 increased in the labeled treatment (Fig. 7). The assigned taxonomy for these OTUs was an unclassified family in the order *Desulfobacterales*, an unclassified family in the order *Coriobacteriales*, and *Methyloversatilis* in the family *Rhodocyclaceae*, respectively. Concerning archaea, the relative abundance of OTU_2071 increased in the labeled treatment (Fig. 7), and it was assigned to an unclassified family in the order *Methanosarcinales*. These differences in abundances between the labeled and unlabeled treatments first appeared on day 38 for all four OTUs, and a clear increase in these OTUs was detected on day 64. These four OTUs were the only species that exhibited this response.

### Combining the results from T-RFLP with cloning/sequencing and time-resolved pyrosequencing

The partial 16S rRNA gene sequences obtained by pyrosequencing were compared with those of the clones. The clones of T-RF1 related to the order *Desulfobacteriales* had more than a 99% identity with sequences in OTU_1012, which meant that the clones were included in the same OTU. Similarly, all the clones of T-RF2 related to *Olsenella* were determined to be members of OTU_3242. We verified the consistency of the results obtained by T-RFLP-cloning and pyrosequencing. The remaining two OTUs, OTU_2694 and OTU_2071, were not the same as any clone obtained in the heavy fraction. In contrast, no OTU corresponded to the two clones related to T-RF1, *i.e.*, SHRa-h17 and SHRa-h36, which belonged to a cluster with *S. propionica* (21).

## Discussion

A methanogenic benzene-degrading enrichment culture was obtained from non-contaminated river sediment in Saitama Prefecture, Japan. In the present study, the production of CH_4_ and CO_2_ started concurrently with the degradation of benzene, while the sum of the amount of CH_4_ and CO_2_ produced did not add up to the original amount of benzene. According to the findings of our previous study (22), biomass yield must be approximately 10% or less of degraded benzene. Thus, the rest of the carbon should have remained as intermediates in the system before the completion of benzene mineralization.

Regarding the community structure, pyrosequencing analysis revealed that the community in the enrichment culture still remained highly complex even though the culture was incubated with benzene as the sole organic carbon source for almost 4 years. Some of the sequences belonged to the same phylotypes as those identified as putative benzene degraders in previous studies using SIP analysis on anaerobic benzene-degrading cultures. The order *Desulfobacterales*, which includes the putative methanogenic benzene degrader Hasda-A (29), accounted for 2.8% of the bacterial reads. Bacteria presumed to be involved in benzene degradation under sulfate-reducing conditions were also detected. *Desulfobacterium* and unclassified species within *Desulfobacteraceae* (24) as well as *Pelotomaculum* within *Peptococcaceae* (16) accounted for 1.4%, 0.5%, and 0.4%, respectively. However, species identified in iron-reducing (19) and nitrate-reducing (18, 20) benzene-degrading cultures by SIP analysis appeared to be in the minority in this culture, *i.e.*, *Desulfobulbaceae* accounted for 0.1% of the bacterial reads, and there were no reads of *Thermincola* within *Peptococcaceae* and reads of *Azoarcus* or *Pelomonas*, while *Pseudomonas* accounted for <0.01% of the reads. On the other hand, the most abundant OTU was assigned to unclassified *Clostridiales*. This OTU was not detected in the SIP experiment, which indicates that this bacterial group was not likely to be involved in the degradation of benzene. The high abundance of this OTU indicates that they have played some role in this consortium, such as degradation of the substrates remaining in the culture from pre-incubation or decomposition of the biomass. Although we previously attempted to detect the presumed intermediates such as toluene, phenol, benzoate, and acetate using GC-FID and high performance liquid chromatography (HPLC), as described in Masumoto *et al.* (22), we were not successful (Masumoto *et al.*, data not published).

Some concerns remain since the abundance of archaea, which was composed mostly of methanogens, was only 3.1% of the whole community. This abundance may be too small because another anaerobic benzene-degrading enrichment culture had the same order of archaeal 16S rRNA gene copies as that of the bacteria (22). This may have been because of bias due to the primer used for pyrosequencing. It may be necessary to analyze important archaea in this community in the future using an alternate method.

The application of DNA-SIP to the samples that completely degraded benzene showed that bacteria related to T-RF1 and T-RF2 incorporated ^13^C derived from benzene. The major clones of T-RF1, which corresponded to OTU_1012 in the pyrosequence analysis, were related to Hasda-A. Among them, clone SHRa-h11 was almost identical (one base mismatch) to the partial sequence of the 16S rRNA gene of clone OR-M2, which was predominantly found in methanogenic enrichment cultures (32). Ulrich and Edwards suggested that clone OR-M2 may initiate the attack on benzene because the 16S r RNA gene of OR-M2 grouped with the aniline- and phenol-degrading bacterium *D. anilini*. Clone OR-M2 belonged to the same cluster as clone SB-21, which was found in a different sulfate-reducing benzene-degrading culture (25). Clones SHRa-h17 and SHRa-h36 were also included in T-RF1, but no OTU related to these clones was found to incorporate ^13^C in the pyrosequence analysis. These bacteria did not incorporate ^13^C as much as the other bacteria detected in this study.

All three clones of T-RF2 that corresponded to OTU_3242 in the pyrosequence analysis belonged to the family *Coriobacteriaceae*. The members of *Coriobacteriaceae* include fermenters, nitrate reducers, and hydrogen producers (2, 10, 34). The closest relative *O. uli* is a strictly anaerobic bacterium that ferments glucose, but does not produce hydrogen (10). As these members of the family *Coriobacteriaceae* do not metabolize aromatic compounds, the clones of T-RF2 could be secondary fermenters or scavengers utilizing dead biomass or secondary metabolites. However, the phylogenetic positions of these clones were very distant from the microbes described, and the family members are physiologically diverse. Therefore, it is difficult to estimate their role in this community on the basis of the information available. Therefore, they could be the benzene degrader.

Both the *Desulfobacterales*- and *Coriobacteriaceae*-related sequences, OTU_1012 and OTU_3242, respectively, incorporated ^13^C on day 38, which was the earliest point during the incubation which we could distinguish in both T-RFLP analysis and pyrosequence analysis. This suggests that they are more likely to be the members of primary benzene degraders.

Other than *Desulfobacterales*- and *Coriobacteriaceae*-related sequences, which correspond to OTU_1012 and OTU_3242, OTU_2694 and OTU_2071 were detected as benzene-derived ^13^C assimilators on day 38. These OTUs were not found in T-RFLP with cloning/sequencing analysis, and this was attributed to their low abundance. OTU_2071 was closely related to acetoclastic methanogens. Methanogens are known to degrade only low-molecular-weight substrates and not aromatic hydrocarbons. It is unlikely that this methanogen was involved in the initial benzene degradation, whereas it is likely that it utilized benzene metabolites such as organic acids and produced CH_4_ by syntrophic association with benzene-degrading bacteria. It was not unexpected that the microorganisms utilizing the benzene metabolites were detected at the same time as the primary benzene degraders because methanogenesis occurred almost concurrently with benzene degradation (Fig. 1 and Fig. 2). OTU_2694 was related to the family *Rhodocyclaceae* in *Betaproteobacteria*. None of its related bacteria have so far been shown to have the capacity to degrade benzene or its possible initial metabolites. Due to the functional versatility of its related bacteria and limited reliability of the relatively short sequence, it is difficult to estimate the role of these bacteria in the community.

The result of the present study, including T-RFLP, cloning/sequencing, and time-resolved analysis based on SIP, indicated that the microbes assimilating ^13^C derived from isotope-labeled benzene are primarily bacteria belonging to *Desulfobacterales* and *Coriobacteriaceae*. The high abundance of *Desulfobacterales*-related bacteria suggests the importance of the bacteria in the degradation of benzene.

## Supplementary Information



## Figures and Tables

**Table 1:** Top 9 bacterial phyla and the families that constituted >1.0% of the bacterial community based on the 16S ribosomal RNA (rRNA) partial gene sequences detected by pyrosequencing.

Phylum	Abundance (%)	Family	Abundance (%)
*Firmicutes*	34.6	Unclassified *Clostridiales*	27.8
		*Clostridiaceae*	2.9
		*Bacillaceae*	1.3
		Others	2.7
*Proteobacteria*	23.6	*Geobacteraceae*	4.8
		*Syntrophaceae*	3.1
		*Helicobacteraceae*	2.9
		Unclassified *Desulfobacterales*	2.7
		*Desulfobacteraceae*	1.9
		*Syntrophorhabdaceae*	1.3
		*Syntrophobacteraceae*	1.1
		Others	5.8
*Chloroflexi*	9.1	*Anaerolinaceae*	3.6
		Clone SHA-20	1.4
		Others	4.1
*Chlorobi*	6.1	*Chlorobiaceae*	4.6
		Clone BSV19	1.1
		Others	0.4
OP8	3.8	Clone SHA-124	1.7
		Others	2.1
*Actinobacteria*	3.4	Mixed culture isolate_koll13	2.2
		Others	1.2
OP1	3.3	Unclassified OP1	3.3
*Bacteroidetes*	2.6	Unclassified *Bacteroidales*	2.6
		Others	0.1
*Spirochaetes*	2.3	*Spirochaetaceae*	1.3
		Others	1.0
Other bacteria	11.2	—	11.2
