# Supplementary material for: Time-Resolved DNA Stable Isotope Probing Links Desulfobacterales- and Coriobacteriaceae-Related Bacteria to Anaerobic Degradation of Benzene under Methanogenic Conditions
Source: Microbes Environ. 2014 Jun 6;29(2):191–9. doi: 10.1264/jsme2.ME13104 (PMC4103526; doi:10.1264/jsme2.ME13104)

Supplementary figure

Article title: Time-resolved DNA stable isotope probing links *Desulfobacterales*- and *Coriobacteriaceae*-related bacteria to anaerobic degradation of benzene under methanogenic conditions

Journal name: Microbes and Environments

Authors: Mana Noguchi*, Futoshi Kurisu, Ikuro Kasuga, Hiroaki Furumai

*Corresponding author: e-mail: noguchi@env.t.u-tokyo.ac.jp; tel: +81-03-5841-6248; fax: +81-03-5841-6244

Figure Legend

Fig. S1 Relative abundance of T-RF1 and T-RF2 in each fraction of ○: unlabeled treatment and ●: labeled treatment on a) day 23, b) day 38, and c) day 64

Fig. S1

First author: Mana Noguchi

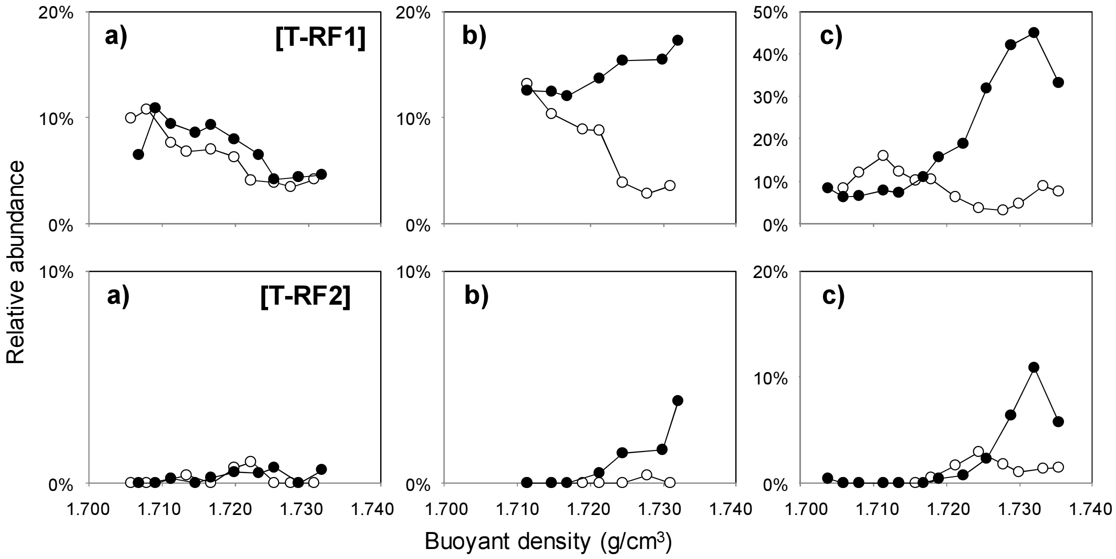

Supplement: Supplementary file 1 [file 29_191_s1.pdf]
